# Luminescence and Formation of Cubic and Hexagonal (K,Rb)_2_SiF_6_:Mn^4+^

**DOI:** 10.1021/acsami.3c13715

**Published:** 2023-12-18

**Authors:** Arnoldus J. van Bunningen, Jur W. de Wit, Sadakazu Wakui, Andries Meijerink

**Affiliations:** † Debye Institute for Nanomaterials Science, Utrecht University, Utrecht 3584 CC, The Netherlands; ‡ Nichia Corporation, 491 Oka, Kaminaka-Cho, Anan-Shi, Tokushima 774-8601, Japan

**Keywords:** red phosphor, Mn^4+^, (K Rb)_2_SiF_6_, zero phonon line, phase transformation

## Abstract

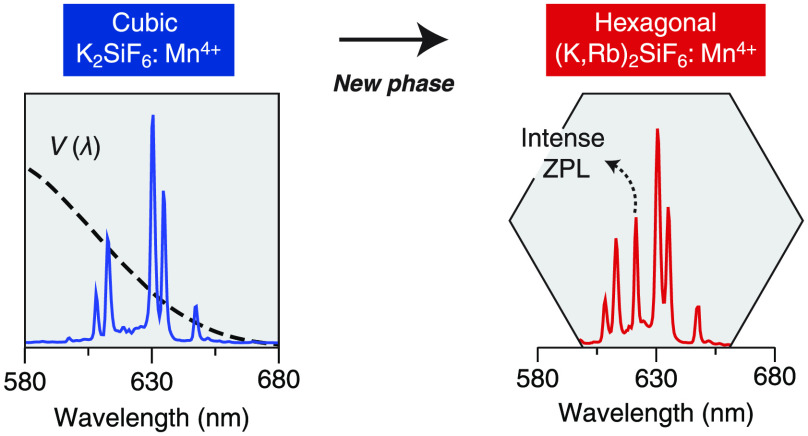

The efficient red-emitting phosphor K_2_SiF_6_:Mn^4+^ (KSF) is widely used for low-power LED applications. The saturated red color and sharp line emission are ideal for application in backlight LEDs for displays. However, the long excited state lifetime lowers the external quantum yield (EQY) at high photon flux, limiting the application in (higher power density) lighting. Here, we report the synthesis of a new crystalline phase: hexagonal (K,Rb)SiF_6_:Mn^4+^ (h-KRSF). Due to the lower local symmetry, the Mn^4+^ emission in this new host material shows a pronounced zero phonon line, which is different from Mn^4+^ in the cubic KSF. The lower symmetry reduces the excited state lifetime, and thus, the loss of EQY under high photon fluxes, and the spectral change also increases the lumen/W output. Temperature-dependent emission and lifetime measurements reveal a high luminescence quenching temperature of ∼500 K, similar to that of KSF. The formation mechanism of h-KRSF was studied *in situ* by measuring the emission spectra of the precipitate in solution over time. Initially, nanocrystalline cubic KRSF (c-KRSF) is formed, which transforms into a microcrystalline hexagonal precipitate with a surprising exponential increase in the transformation rate with time. The stability of the new phase was studied by temperature-dependent XRD, and an irreversible transition back to the cubic phase was seen upon heating to temperatures above 200 ^°^C.

## Introduction

In the field of lighting and displays, the discovery of the blue-light-emitting diode (LED) marks the beginning of a revolution. Converting a part of the blue LED output to longer wavelength light (green, yellow, orange, or red) by a luminescent material (a phosphor) makes it possible to realize compact and efficient white light sources with a great flexibility in spectral distribution. The desired characteristics of a phosphor depend on the application. Lighting requires white LEDs (wLEDs) with a high efficacy (lumen/W output) and a high color rendering index (CRI). The high brightness of wLEDs can be realized only using phosphors with emitters that have a high turnover rate (photons/s). In addition, the luminescence quenching temperature has to be high as the device locally heats up to 150 ^°^C. In displays, brightness is lower, and this sets less stringent requirements on turnover rate and stability. However, phosphors with saturated colors with emission at specific wavelengths are preferred to extend the color gamut while remaining efficient.

A successful red-emitting phosphor, especially for displays, is K_2_SiF_6_:Mn^4+^ (KSF). The Mn^4+^ ion has the 3d^3^ configuration, and in fluorides, it shows a sharp line emission around 620 nm due to vibronic ^2^E → ^4^A_2_ transitions. The narrow spectral distribution is ideal for display applications. The luminescence of KSF was reported already back in 1973, but it was not until 2006 that its potential in LED lighting and displays was realized.^1,2^ The spectral properties of KSF are superior to those of other red LED phosphors such as CaAlSiN_3_:Eu^2+^ (CASN). The broad band red Eu^2+^ emission extends toward the NIR, where the eye sensitivity is low. This reduces the lumen/W output. The narrow line emission around 620 nm of Mn^4+^ helps to extend the color gamut in displays. Unfortunately, for higher power applications, KSF is less suitable, because the long emission lifetime (∼10 ms) for the parity- and spin-forbidden ^2^E → ^4^A_2_ transition limits the turnover rate to ∼100 photons/s per Mn^4+^ ion and, thus, lowers the external quantum yield (EQY) at higher photon fluxes.

The popular KSF phosphor has a cubic crystal structure, and the Mn^4+^ ion is in a centrosymmetric octahedral coordination of fluoride ions. The inversion symmetry makes the parity selection rule strict, and it can only be lifted by coupling with odd-parity vibrations. As a result, the sharp emission lines observed are vibronic lines corresponding to ungerade vibrations that induce odd-parity crystal field components. The strictly forbidden zero-phonon line (ZPL) is not observed, and the luminescence lifetime of the ^2^E state is long. Later, other Mn^4+^-doped fluoride hosts were found with hexagonal or trigonal crystal structures.^3−5^ The lower local symmetry for the Mn^4+^ ion results in the appearance of a ZPL (induced by static odd-parity crystal field components) in addition to vibronic lines.^6^ The emission lifetime for Mn^4+^ in these hosts is shorter, and the higher eye sensitivity at the ZPL wavelength is also beneficial for the efficacy. Unfortunately, for all of these hosts, the luminescence quenching temperature and/or stability were low, and they could not replace KSF, in spite of the superior spectral properties.

There has been a search for Mn^4+^ phosphors similar to KSF but with a lower symmetry crystal structure to decrease the lifetime and induce a ZPL. Such a phosphor is reported here: hexagonal (K,Rb)SiF_6_:Mn^4+^ (h-KRSF:Mn^4+^). Normally, K_2_SiF_6_ and Rb_2_SiF_6_, as well as their solid solutions form a cubic phase. Yet, here we show that under specific reaction conditions, the mixed solid solutions can form in a stable hexagonal phase. Interestingly, the existence of a hexagonal crystal structure for KSF or KRSF has sporadically been reported. The crystal structure was described by Kolditz and Preiss in 1963,^7^ referring back to earlier reports from 1904.^8^ In the mineralogy of fumaroles, grains of 0.3 mm have been reported with the chemical formula K_2_SiF_6_ and hexagonal symmetry,^9^ while in 1952, hexagonal KSF was found when analyzing chemicals of a decommissioned chimney that was used to drain sulfuric acid and hydrogen fluoride gases.^10^ There are also a few recent examples of the hexagonal form of KSF. In 2015, the luminescence of KSF:Mn^4+^ was measured at increasing pressure. Between 9 and 13 kbar, a strong ZPL arises that does not disappear after decompression. This could be due to the formation of nanocrystalline hexagonal KSF, but XRD measurements after decompression did not indicate a cubic-to-hexagonal phase transformation.^11^ In 2014, hexagonal KSF:Mn^4+^ was synthesized, but no luminescence was observed.^12^ So far, no reports have been made that measured and verified Mn^4+^ in hexagonal KSF. For h-KRSF, there is one patent that reports the existence and luminescence of this phase and describes the synthesis of KRSF:Mn^4+^ as a phosphor.^13^

In this paper, we describe the reproducible synthesis of hexagonal KRSF:Mn^4+^. We report the improved luminescence properties induced by the lower site symmetry for Mn^4+^ in the hexagonal phase and evaluate the advantageous properties such as a shorter luminescence decay time and a strong ZPL increasing the efficacy of the phosphor. We follow the formation of h-KRSF by measuring the Mn^4+^ emission to probe the phase transition from cubic to hexagonal and show how after a long induction period, h-KRSF starts to form, and the transformation rate of h-KRSF increases exponentially with time. Finally, we determined the temperature stability of h-KRSF by measuring the back transformation to the cubic KRSF via temperature-dependent XRD and luminescence measurements.

## Methods

### Synthesis

The synthesis procedure for KRSF is inspired by previously reported methods for KSF. As a Mn precursor, K_2_MnF_6_ was used. As this is not commercially available because of the low stability, it was synthesized as described by Roesky.^14^ Other chemicals used were 48% HF and 30% H_2_SiF_6_ solutions from Sigma-Aldrich, KHF_2_ from Strem Chemicals, and RbF from Chempur.

For the typical synthesis of KRbSiF_6_:0.5% Mn^4+^, 12 mg K_2_MnF_6_, 0.391 g KHF_2_, and 0.523 g RbF were dissolved in 1.5 mL aqueous HF (48 vol %). In a second beaker, 1.5 mL of aqueous 30 wt % H_2_SiF_6_ was combined with 5 mL of 48% HF. Upon combining the two solutions, some turbidity was observed. To regain full dissolution of all precursors, ∼20 mL aqueous HF was added until a clear solution was obtained. This solution was added to four times the volume of ethanol (EtOH) (∼100 mL). No precipitate was visible by naked eye, but under illumination with a hand-held violet laser (405 nm), the solution showed red luminescence. This indicates the formation of nanosized KRSF particles. The aqueous EtOH solution was left to evaporate for 2 days to a week in the fume hood. The amount of precipitate gradually increases during evaporation. After all the liquid evaporated, the solid material was washed with 3% H_2_O_2_ aqueous solution and subsequently with EtOH, after which it was dried at 100 °C for 1–2 h. The hexagonal KRSF (h-KRSF) synthesized through this procedure contained 20–50 mol % of Rb. The K, Rb, and Mn concentrations in the samples discussed below were measured with ICP-OES, and the values can be found in Section S4.

For comparison, cubic KRSF (c-KRSF) was synthesized. Two different methods were employed. One involved immediate separation by decantation of the precipitate formed directly after the addition of H_2_SiF_6_ in the synthesis method described above. The second method was heating the hexagonal KRSF to 400 °C for 30 min.

### Characterization

The powders were examined using powder X-ray diffraction to determine the phase purity. A Bruker D2 PHASER X-ray diffractometer with a Co source (λ_Kα_ = 1.7902 Å) was used at 30 kV operating voltage and 10 mA current. The temperature-dependent X-ray diffraction measurements were performed with a Malvern Panalytical Aeris Research diffractometer equipped with an Anton Paar BTS 500 heating stage and a Cu K_α_ (λ_Kα_ = 1.5418) radiation source.

The K, Rb, and Mn concentrations in the phosphors were examined with inductively coupled plasma optical emission spectroscopy (ICP-OES). All measurements were performed on a PerkinElmer Optima 8300DV spectrometer (Mn λ_em_ = 257.610 nm, Rb λ_em_ = 780.023 nm, and K λ_em_ = 766.490 nm). Aqua regia was used to dissolve the phosphors.

### Optical Spectroscopy

Photoluminescence (PL) and PL excitation (PLE) spectra were recorded using an Edinburgh Instruments FLS 920 spectrofluorometer equipped with a 450 W Xe lamp as the excitation source and a Hamamatsu R928 photomultiplier tube (PMT) detector. PL decay curves were recorded using a tunable optical paramagnetic oscillator (OPO) Opotek Opolette HE 355II giving ∼1–5 mJ pulses in the visible or near-infrared (pulse width: 10 ns; repetition rate: 20 Hz) as excitation source and the multichannel scaling (MCS) capabilities included in the Edinburgh spectrofluorometer. For temperature-dependent studies, a temperature-controlled stage from Linkam Scientific (THMS600) was built in the spectrofluorometer for measurements in a −190 to 450 °C temperature range. Measurements down to 4 K were performed with an Oxford Instruments liquid-He cold-finger cryostat.

The *in situ* monitoring of the cubic-to-hexagonal phase transformation was performed with a custom-built optical setup. In short, the beaker containing the reaction mixture was illuminated from above with an OBIS LX 445 nm, 45 mW laser with a fiber pigtail output. An AvaSpec-HSC 1024 × 58 TEC-EVO CCD spectrometer equipped with an optical fiber and a 472 nm long-pass filter was used to collect the red emission on the side of the beaker to measure emission spectra at regular time intervals during the formation (for up to several days).

### DFT Calculations

To assess the stability of the cubic vs. hexagonal phase for KSF, RSF, and KRSF, first-principles total-energy calculations^15^ were performed based on density functional theory (DFT)^16,17^ using the projector augmented wave (PAW) as implemented in the Vienna ab initio simulation package.^18,19^ Frozen core approximation was combined with PAW, and the valence electron configurations are 3s^2^3p^6^4s^1^ for K, 4s^2^4p^6^5s^1^ for Rb, 3s^2^3p^2^ for Si, and 2s^2^2p^5^ for F. Exchange and correlation were treated with the generalized gradient approximation.^20^ The wave functions were expanded in a plane-wave basis set with a kinetic energy cut-off of 600 eV. 8 × 8 × 8 and 6 × 6 × 4 Monkhorst-Pack *k*-point meshes were used for the integration in *k* space in the Brillouin zone for the cubic and hexagonal unit cells, respectively. The structural optimizations were performed until each component of the interatomic force became less than 1.0 × 10^–3^ eV/Å.

## Results and Discussion

### Phase Identification

**Figure 1:**
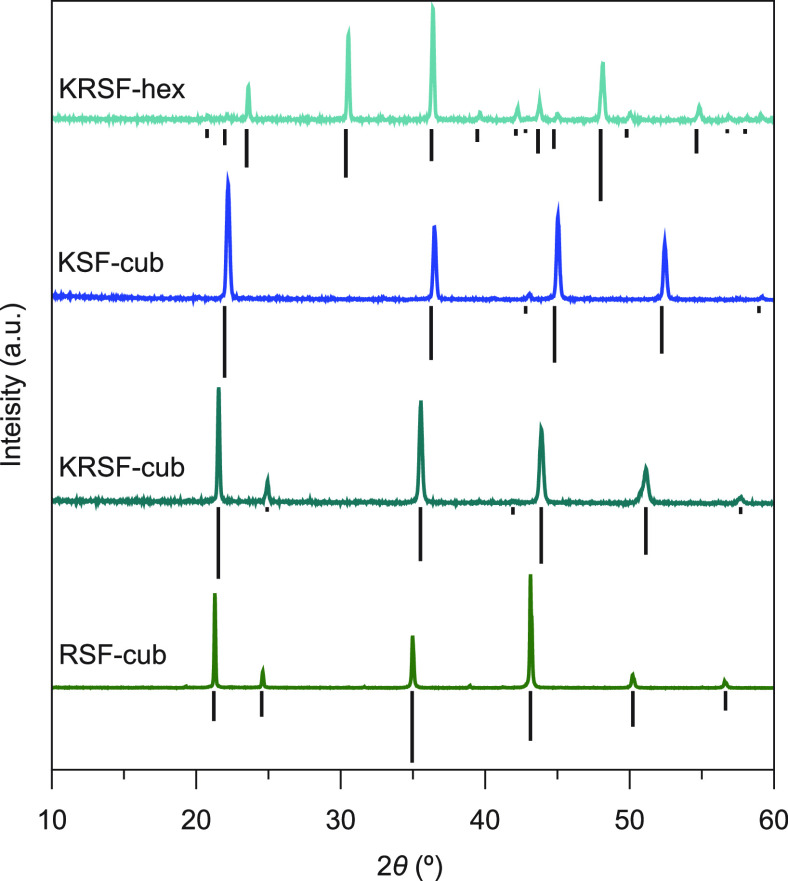
| X-ray diffractogram of Mn^4+^-doped fluorides. From top to bottom, the diffractograms of hexagonal KRbSiF_6_, cubic K_2_SiF_6_, cubic KRbSiF_6_, and cubic Rb_2_SiF_6_ doped with 0.5% Mn^4+^ are shown. Below the experimental patterns, the reference patterns are plotted.^9,21,22^ Note that these diffractograms are recorded using a Co X-ray source.

To investigate the crystal structure and phase purity of the different materials, after synthesis, the dry powders were checked by measuring the X-ray diffractograms. In Figure 1, the diffractograms of the different microcrystalline powders are shown with their respective references underneath.

In Figure 1, we can see that for all samples, there is good agreement with the reference diffraction patterns. This shows that the different synthesis methods result in phase-pure crystalline materials. For cubic KSF and RSF, the crystal structure is well established, and the reference diffractograms are well known. For c-KRSF, the diffraction lines are at angles in between KSF and RSF, as expected for a solid solution. A good agreement with the experimentally observed positions of diffraction lines was obtained by assuming an increase of 2% in lattice distances compared with the KSF reference. A slight increase is expected by the replacement of K by Rb as the ionic radius of Rb^+^ (1.72 Å) is larger than that of K^+^ (1.64 Å), causing a small expansion of the unit cell.^23^

The reference pattern of h-KRSF is based on an earlier work on hexagonal KSF. In ref. (9), the XRD pattern for h-KSF is reported and used to derive lattice parameters *a* = 5.67 and *c* = 9.24 and identify two different sites for the K^+^ ion, a smaller M1 and a larger M2 site. The diffraction pattern obtained here for KRSF is very similar. A good match is obtained for slightly larger lattice parameters *a* = 5.78 and *c* = 9.42, providing convincing evidence for the formation of hexagonal KRbSiF_6_:Mn^4+^. The powder XRD data do not allow us to distinguish between the ordering of Rb^+^ and K^+^ on the M1 and M2 sites. It will be interesting to obtain high-quality single crystal data to obtain information on site occupation in the mixed crystal.

**Figure 2:**
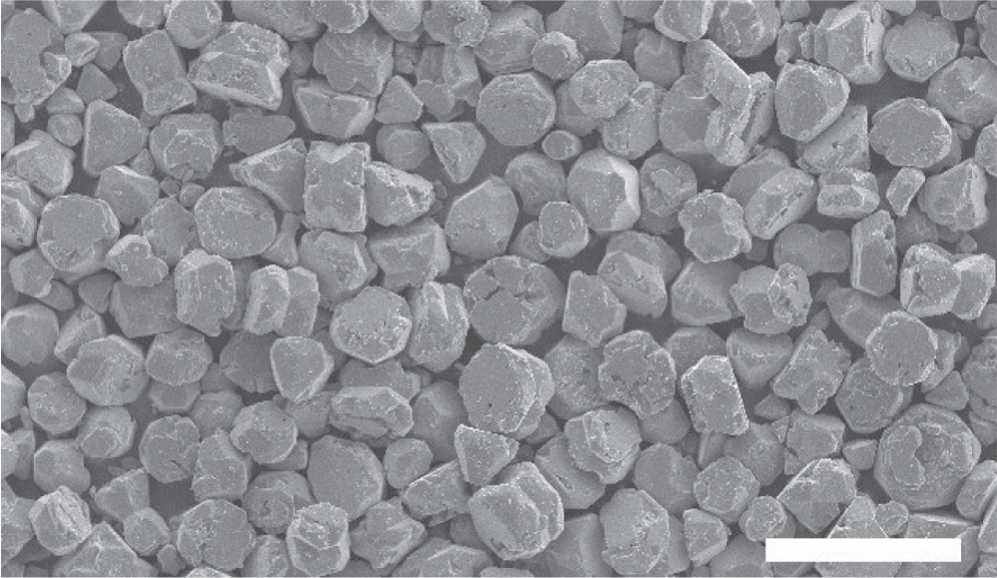
SEM image of synthesized h-KRSF:1.8% Mn^4+^. Scale bar at the bottom is 100 μm. The average particle size is ∼30 μm.

To evaluate the particle size and particle size distribution, we made SEM images of the final product. The SEM image in Figure 2 shows that the synthesis procedure used results in a homogeneous particle size distribution with an average particle size of ∼30 μm.

### Optical Properties

**Figure 3:**
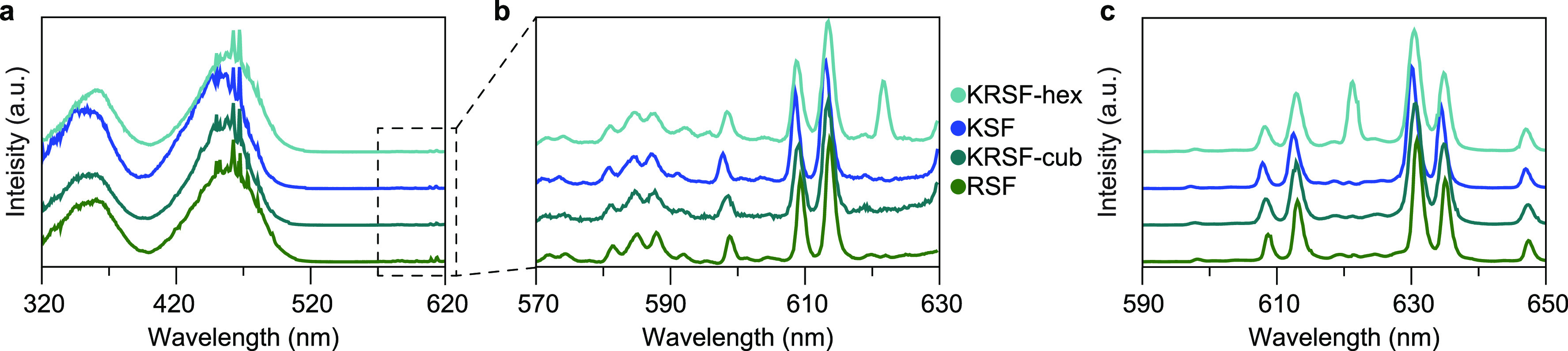
Photoluminescence excitation (PLE) and photoluminescence (PL) spectra of h-KRSF and cubic M_2_SiF_6_ (M = K and/or Rb) doped with 0.1–0.5% Mn^4+^. (a) PLE spectra recorded at λ_em_ = 630 nm that show two spin-allowed ^4^A_2_ →^4^T_2_ and ^4^A_2_ → ^4^T_1_ excitation bands centered at 460 and 360 nm, respectively. (b) PLE spectra were recorded for 648 nm emission, showing the spin-forbidden, vibronic ^4^A_2_ → ^2^T_1_, and ^2^E excitation lines. Multiple vibronic excitations are visible for both ^4^A_2_→ ^2^E excitation (range 598–619 nm) and the ^4^A_2_ → ^2^T_1_ excitation (570–596 nm). (c) PL spectra of the ^2^E → ^4^A_2_ transition excited at 450 nm. Only for the h-KRSF samples is the ZPL clearly present at 621.5 nm, in excitation and emission.

To study the optical properties of Mn^4+^ in the new h-KRSF, both PL and PLE spectra were measured for low-doped samples (0.1–0.5% Mn^4+^). For comparison, the spectra of Mn^4+^ in cubic KRSF, KSF, and RSF were measured as well. In Figure 3, it is observed that all the PLE spectra have two relatively strong and broad excitation bands around 360 and 460 nm. The 460 nm band shows some sharp lines around 470 nm. These can be ascribed to Xe-lamp lines that are visible in spite of correcting the spectra for variations in the Xe-lamp intensity. A zoom-in for the area between 560 and 625 nm shows a multitude of weak and narrow excitation lines. The PLE spectra of the four samples are very similar to one exception: there is a sharp extra peak at 621.5 nm for Mn^4+^ in h-KRSF. In the PL spectra (Figure 3c) again, all spectra are very similar showing sharp emission lines at the same positions, with small shifts of ∼0.5 nm to longer wavelengths from KSF to RSF. Again there is one exception: an extra peak at 621.5 nm for Mn^4+^ in the hexagonal form of KRSF.

Based on the Tanabe–Sugano diagram for 3d^3^ ions in octahedral symmetry, the excitation bands at 360 and 460 nm in the PLE spectra are assigned to the ^4^A_2_ → ^4^T_1_ and ^4^A_2_ → ^4^T_2_ transitions, respectively. In the zoom-in spectra, Figure 3b, the peaks observed from 560 to 595 nm are assigned to the vibronic lines of the ^4^A_2_ → ^2^T_1_ transition and from 600 to 625 nm to vibronic excitation lines of the ^4^A_2_ → ^2^E transition in the cubic modifications. For Mn^4+^ in inversion symmetry, all 3d^3^ → 3d^3^ transitions are parity forbidden, and coupling with odd-parity vibrations is required to partly lift the parity selection rule, resulting in the observation of vibronic excitation and emission lines. In h-KRSF, the Mn^4+^ ion is in a site with lower symmetry, and static odd-parity crystal field components allow for breaking the parity selection rule. As a result, also the purely electronic zero-phonon transition can be observed. For the ^4^A_2_ → ^2^E transition in h-KRSF, this zero-phonon line (ZPL) is at 621.5 nm and is identical in the excitation and emission spectra. The positions of the vibronic emission lines in KSF (Figure 3c) are 597, 608, 613, 630, 635, and 648 nm, in agreement with earlier reports. The lines at 630, 635, and 648 nm are Stokes vibronic lines due to coupling with ν_6_, ν_4_, and ν_3_ vibrations. The lines at 597, 608, and 613 nm are anti-Stokes vibronics at the same energy differences from the ZPL (that is hardly observed, except for h-KRSF) as the Stokes lines.

The change in local symmetry around the tetravalent ion in cubic KSF to hexagonal KSF is key for understanding the appearance of the ZPL. In the cubic phase, the Si^4+^ atom (or the Mn^4+^) is symmetrically surrounded by six equidistant fluorine ligands at 1.677 Å. In ref. (9), a Rietveld refinement on the diffraction pattern of the hexagonal phase of KSF shows that there is a slight distortion of the octahedron: three ligands are at a distance of 1.681 Å while the others are at a distance of 1.688 Å.^9,21^ A similar deviation from inversion symmetry for Mn^4+^ can be expected in h-KRSF and explains why for the hexagonal phase a zero phonon line is observed and not for the cubic phases. Again, it will be interesting to obtain single crystal data to determine the deviation from octahedral coordination for the [MF_6_]^2–^ units in the K^+^/Rb^+^ mixed crystal and compare this with other Mn^4+^ fluoride hosts where a ZPL is observed.

The enhanced ZPL is beneficial for the performance. The additional emission at ∼620 nm where the eye sensitivity is higher, increases the efficacy. The luminous response function has its maximum at 550 nm and drops to 1% of the maximum at 680 nm. A higher fraction of the emission spectrum toward longer wavelengths reduces the efficacy. If we compare c-KRSF to h-KRSF, a smaller fraction of the emission is from the Stokes emission lines of 630, 636, and 648 nm. The additional emission intensity at ∼620 nm results in an efficacy increase of 2.9% for h-KRSF compared with c-KRSF (see Section S1 and Figure S1). In addition, deviation from the inversion symmetry also increases the ^4^A_2_ → ^4^T_2_ absorption strength for the blue excitation wavelength at 450 nm as a result of relaxation of the parity selection rule. The increased absorption strength at 450 nm is experimentally observed to be ∼34% by comparing the emission intensity of c-KRSF with h-KRSF under the same excitation intensity (see Section S1 and Figures S1 and S2). To evaluate the efficiency of the new h-KRSF, phosphor quantum yield measurements were done. A sample with 1.8 mol % Mn incorporated had an internal quantum yield of 91%. We consider this value to be very high as little effort was put into optimizing the synthesis.

For practical applications, a wLED phosphor needs to be resilient to high temperatures and a humid atmosphere. To test the stability, the luminescence of h-KRSF was measured after synthesis, and this was compared to the luminescence after 48 h exposure to 85% humidity at 85 °C. A KSF phosphor was measured simultaneously. For the h-KRSF, a decrease in luminescence of 16% was seen, which is considerably worse than that of the KSF, which showed a loss of 1–2%. The relatively fast degradation of h-KRSF compared with KSF is attributed to the incorporation of Rb. Rb compounds tend to be more hygroscopic than K compounds, thus, enhancing the degradation.^24^ For practical application, the stability needs to be improved, e.g., by postsynthesis treatment, overcoating, and/or encapsulation in a protective matrix using strategies that are also explored for KSF.^25,26^ Reducing the Rb content from 50% to 20% (a Rb concentration for which the hexagonal phase can still be obtained, *vide infra*) may also enhance the stability. Furthermore, optimization is required to explore the potential of h-KRSF as a new LED phosphor. An initial test with h-KRSF phosphor in a w-LED shows promising results with a performance that is similar to that of a wLED with KSF (see Section S2).

### Concentration-Dependent Luminescence

The 450 nm absorption by Mn^4+^ in the ^4^A_2_ → ^4^T_1_ absorption band involves a spin-allowed, but parity-forbidden transition. As discussed above, the deviation from inversion symmetry in h-KRSF is expected to make the absorption stronger than that in c-KRSF or KSF, but this absorption is still much weaker than for fully allowed transitions such as the 4f^*n*^ → 4f^*n*–1^5d transition in Ce^3+^ or Eu^2+^. A high Mn^4+^ concentration is, thus, beneficial for reducing the amount of phosphor required to absorb sufficient blue LED light in a wLED. At the same time, a high dopant concentration can lead to concentration quenching. Energy transfer between neighboring ions will cause migration of the excitation energy over the dopant sublattice. Especially above the percolation point (where a 3D connected lattice of dopant ions is realized), the migrating excitation energy can probe a large volume in which there is a high probability to encounter a defect or impurity quenching site causing concentration quenching. Investigating the concentration dependence of the luminescence efficiency is therefore important, and a concentration series of h-KRSF:Mn^4+^*x*% (*x* = 0.1–10) was synthesized (see Section S3 for the XRD patterns). It is important to realize that the fraction of Mn^4+^ in the synthesis mixture is not the same as the fraction incorporated in the h-KRSF. Indeed, after evaporating the EtOH out of the reaction mixture, darker colored spots are visible within the dry powder. Washing with H_2_O_2_ removes these spots. Probably these spots were compounds with a high concentration of Mn that dissolve in H_2_O_2_.^27^ This also means that the fraction of Mn^4+^ incorporated in h-KRSF is lower than the nominal concentration. To check the actual Mn concentration, inductively coupled plasma optical emission spectroscopy (ICP-OES) measurements were done. The measurements show that 16–60% of the added Mn is actually incorporated (see Section S4). The concentrations mentioned below always refer to actual concentrations in the phosphors, as determined with ICP-OES.

**Figure 4:**
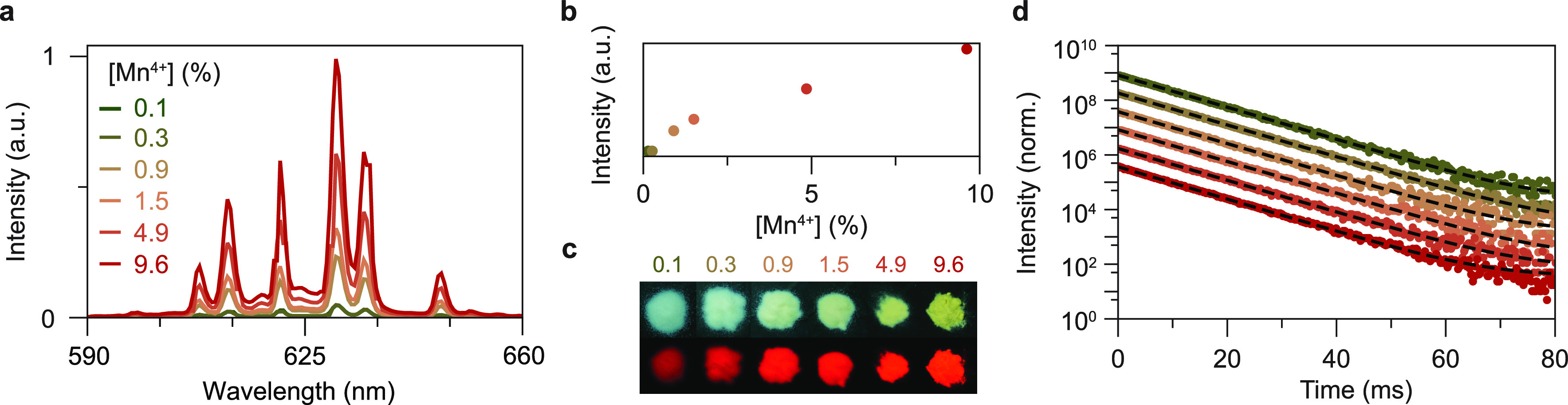
Optical properties of h-KRSF at different Mn^4+^-doping concentrations. (a) Emission spectra of h-KRSF for different doping concentrations that range from 0.1–9.6 mol % with respect to Si^4+^ (λ_exc_ = 450 nm). All samples are diluted 10× (wt %) with BaSO_4_. (b) Integrated intensity of the emission spectra in panel a. The sublinear increase in emission intensity with [Mn^4+^] is ascribed to saturation in the absorption of the blue excitation light. (c) Photographs of the undiluted KRSF samples under flashlight (top row) and 360 nm UV (bottom row) illumination. (d) PL decay curves of the same samples as in panel a for nanosecond pulsed excitation at 450 nm and emission at 630 nm. An offset between the data sets was added for clarity. The dashed lines show the single-exponential fit to the experimental data.

To study the concentration-dependent optical properties, both emission spectra and luminescence decay curves were measured for samples with Mn^4+^ concentrations varying between 0.1 and 10 mol %. In Figure 4a, the emission spectra of samples with different Mn concentrations are shown under 450 nm excitation. The samples were diluted 10× (wt %) with optically inactive BaSO_4_ to limit the path length of light through the h-KRSF phosphor and reduce saturation effects in blue light absorption. It can be seen that the intensity increases with an increasing Mn concentration. The integrated intensities as a function of Mn^4+^ concentration (Figure 4b) show a rapid increase at low concentrations (up to 1% Mn^4+^), after which it levels off. This nonlinear increase at high dopant concentrations has been observed before and is explained by saturation of blue light absorption. The integrated emission intensities of the undiluted phosphors show an even stronger leveling off with increasing Mn^4+^ concentration (Section S5). As the Mn^4+^ concentration increases, a substantial part of the blue light is absorbed, and the fraction of absorbed light no longer increases linearly with Mn^4+^ concentration, as is also evident from Lambert–Beers’ law. Only for a low value of ε*cl* (molar extinction coefficient × concentration × path length), the fraction of absorbed light increases linearly with concentration. This makes it difficult to determine if concentration quenching occurs based on concentration-dependent emission intensities.

A better method to study concentration quenching is by measuring luminescence lifetimes. In the case of nonradiative loss processes as a result of concentration quenching, the emission lifetime will decrease. Luminescence decay curves of the 630 nm emission after pulsed 450 nm excitation are shown in Figure 4d. A single exponential decay is observed for all concentrations, and the decay times are constant ∼6.2 ms. The single exponential decay curves and constant decay time indicate that no concentration quenching occurs up to at least 10% Mn^4+^.

### Temperature-Dependent Luminescence

**Figure 5:**
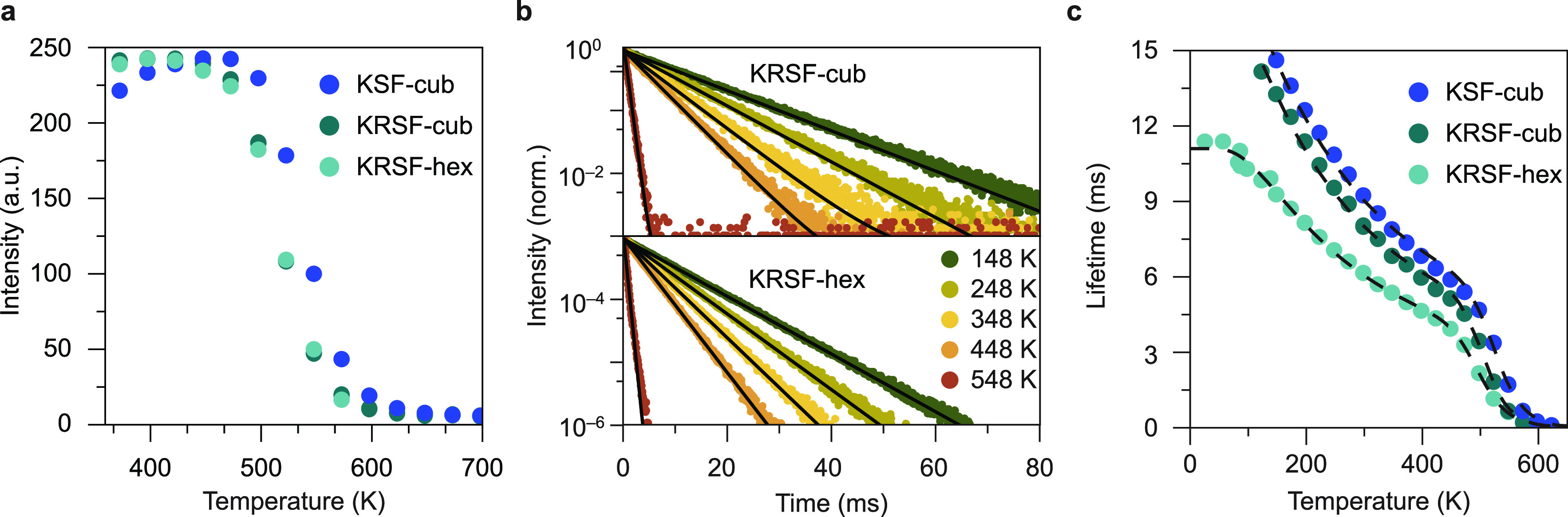
Temperature-dependent optical properties of KSF and KRSF. (a) Integrated emission intensity as a function of temperature for cubic KSF and cubic and hexagonal KRSF doped with 0.1% Mn^4+^. (b) PL decay measurements of Mn^4+^ emission in cubic (top) and hexagonal (bottom) KRSF, single-exponential fits to the data are shown in black. (c) Emission lifetime of the Mn^4+^ emission as a function of temperature in cubic KSF, c-KRSF, and h-KRSF. All samples were excited at 450 nm, and luminescence decay was recorded at 631 nm (KSF and c-KRSF) or 621 nm (h-KRSF).

The temperature stability of the luminescence is an important aspect of wLED phosphors. Heat is generated by the LED chip and also by heat dissipation inherent to the conversion of a higher energy blue photon to green or red photons. The local temperature of a phosphor in wLEDs can easily reach 150 °C. The thermal quenching behavior is therefore crucial. Indeed, previously Mn^4+^-doped fluorides have been found where the lower local symmetry also resulted in the desired observation of a strong ZPL and shorter emission lifetime, but the poor thermal quenching behavior made these phosphors unfit for application in wLEDs.^3,5,28^ The thermal quenching behavior of h-KRSF:0.1% Mn^4+^ was, therefore, measured and compared with those of cubic KRSF:0.1% Mn^4+^ and KSF:0.5% Mn^4+^. The temperature dependence of the integrated emission intensities in the relevant high temperature region 373–700 K is shown in Figure 5a. The corresponding emission spectra at different temperatures of the three samples are shown in Section S6. When the temperature increases, the emission intensity remains constant until 450 K, above which it starts to decrease.

Measuring emission intensity as a function of temperature to probe thermal quenching can be complicated by intensity variations not related to thermal quenching, for example, when the oscillator strength of the absorption transition is temperature dependent. In addition, practical aspects, such as changes in alignment, collection efficiency, or excitation source intensity, can give rise to intensity variations not related to thermal quenching. A fast and reliable method to determine the thermal quenching temperature is to measure the emission lifetime as a function of temperature. As nonradiative decay sets in, the emission lifetime shortens because the lifetime is the inverse of the sum of radiative and nonradiative decay rates. Therefore, lifetimes were also measured as a function of temperature for h-KRSF:0.1% Mn^4+^, c-KRSF:0.1% Mn^4+^, and KSF:0.5%: Mn^4+^ and are shown in Figure 5b. All the decay curves are single exponential. The lifetimes of the Mn^4+^ emission in the three different host lattices are shown as a function of temperature in Figure 5c. For all three host matrices, it can be seen that the lifetime decreases slowly up until 450–480 K after which the lifetime drops sharply, consistent with the temperature-dependent intensity measurements.

Before discussing the luminescence quenching temperature, it is interesting to discuss differences in lifetimes for Mn^4+^ emission in the three compounds: the lifetime is longer for KSF and c-KRSF than that for h-KRSF. As discussed above, the perfect octahedral coordination in the two cubic lattices imposes a strict parity selection rule. This does not only prevent the observation of a ZPL but also reduces the overall transition probability as the ZPL transition is forbidden. The room temperature emission lifetime is ∼6 ms for Mn^4+^ in h-KRSF vs. ∼8 ms in the cubic lattices. The shorter lifetime in h-KRSF is beneficial for application in wLEDs. As mentioned in Introduction, the long emission lifetime is a limiting factor in the total light output and prevents the application of KSF in high-brightness wLEDs. The 25% shorter lifetime helps to improve the performance of h-KRSF in higher brightness sources although the lifetime is still long compared to that for emission in other wLED phosphors, relying on d–f emission from Ce^3+^ (∼40–80 ns) or Eu^2+^ (∼1–2 μs).

In Figure 5a, it is observed that the luminescence intensity is constant until 450 K, while the lifetime decreases gradually with the temperature between 100 and 400 K (Figure 5c). This is an indication that the change in emission lifetime is not caused by temperature quenching. This is generally observed for the ^2^E emission of Mn^4+^ and explained by an increase in vibronic transition probabilities induced by a higher phonon occupation number *n*. It is well-established that the transition probability for Stokes vibronics scales with (*n* + 1) and anti-Stokes vibronics with *n*.^28^ The corresponding change in radiative lifetime as a function of temperature is described by
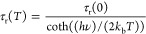
1

Here, τ_r_(*T*) is the radiative lifetime at temperature *T* (in K), *h*ν is the effective phonon energy, and *k*_b_ is the Boltzmann constant. This equation describes the emission lifetime before temperature quenching sets in at 450 K. Temperature quenching for Mn^4+^ has been shown to occur via the ^4^T_2_ state with an activation energy Δ*E*. Together with temperature dependence for the radiative decay time τ_r_(*T*) from eq 1, the expression for the lifetime as a function of temperature is
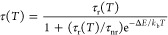
2 where τ_nr_ is the nonradiative decay time, which is typically in the order of picoseconds, the time scale of vibrations. We can now use eqs 1 and 2 to find the quenching temperature *T*_50_, defined as the temperature at which τ(*T*) = (1/2)τ_r_(*T*) . The *T*_50_ temperatures determined in this way for KSF, c-KRSF, and h-KRSF were found to be 530, 510, and 503 K, respectively. All temperatures are sufficiently high to prevent thermal quenching in wLEDs. There is a small decrease in *T*_50_ from KSF to KRSF. The thermal luminescence quenching mechanism has been shown to occur by thermal crossover from the ^2^E state to the ^4^A_2_ state via the ^4^T_2_ state. The lower the energy of the ^4^T_2_ state, the lower the quenching temperature will be. The slightly lower *T*_50_ values for KRSF are consistent with a small red shift (from 452 nm in KSF to ∼458 nm in c-KRSF) of the ^4^T_2_ excitation band. The small redshift may be related to slightly larger distances to the F^–^ ligands in compounds with increasing Rb content, which lowers the crystal field splitting.^28^

### Formation Mechanism

**Figure 6:**
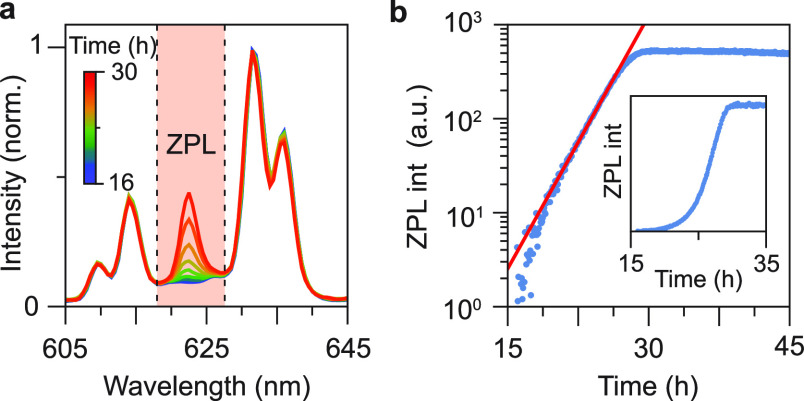
*In situ* emission spectroscopy measurements to follow h-KRSF:Mn^4+^ formation. (a) A selection of emission spectra recorded between 16 and 30 h after pouring the precursor mixture into EtOH. During this time, the ZPL intensity increases strongly, indicating the transition from c-KRSF to h-KRSF. (b) Logarithmic plot of the integrated emission intensity of the ZPL peak, corrected for “background” signal as recorded before 16 h. The integration limits are indicated by the highlighted area in panel a. A single exponential function is fitted to the data between 16 and 26 h after mixing and is plotted in red. The inset shows the corrected ZPL intensity between 15 and 35 h in a linear plot.

The formation mechanism of h-KRSF is intriguing. The method was found serendipitously: the addition of extra aqueous HF to dissolve the initial precipitate followed by the addition of EtOH was meant to precipitate a random mixed phase Rb/K system to investigate the role of disorder in a more distant cation (K/Rb) coordination sphere on the Mn^4+^ luminescence. Interestingly, the absence of a ZPL in c-KRSF shows that deviations from inversion symmetry caused by disorder in the second (K/Rb) coordination sphere are too small to effectively relax the parity selection rule, as was the original goal of the research project. When the EtOH addition did not result in precipitation, the solution was left to evaporate for several days, and a new h-KRSF phase was found. It is interesting to obtain better insight into the formation mechanism of h-KRSF. Therefore, to follow the formation of h-KRSF, emission spectra were recorded during the evaporation process. A 445 nm laser was used to illuminate the reaction beaker (as shown in Section S7), while the emission spectra were recorded at regular time intervals over a period of days by a simple fiber-coupled CCD spectrometer. The results are listed in Figure 6. Immediately after pouring the reaction mixture in EtOH, the solution shows emission spectra typical of the cubic phase with vibronic Stokes and anti-Stokes emission lines, but no ZPL. No precipitation is observed, however, blue excitation showed that much of the c-KRSF immediately concentrated on the lower part of the beaker. The presence of the typical Mn^4+^ spectrum without a ZPL in the initially formed clear solution indicates that nanocrystalline c-KRSF is formed, and based on the higher concentration at the bottom of the beaker, the particle size is estimated to be 50–100 nm. The characterization and optical properties of nanocrystalline KRSF (and KSF) deserve further study but are beyond the scope of this work. Stabilizing the KRSF nanocrystals may be interesting for applications where nanocrystalline KSF offers advantages over the conventional microcrystalline material.

To follow the transformation from cubic to hexagonal KRSF, the emission spectra as recorded over time are shown in Figure 6. The formation of h-KRSF is probed by monitoring the intensity of the ZPL. No ZPL is present in the cubic phase, and by integrating the 618–627 nm range, the ZPL intensity is measured by subtracting the background measured in spectra recorded immediately after the addition to EtOH. The integrated ZPL intensity increases over time and shows a peculiar time dependence. There is a delay in the formation, and only after ∼15 h the transformation to h-KRSF starts and a small ZPL appears. The relative intensity of the ZPL increases, first slowly and then rapidly until all c-KRSF is transformed into h-KRSF. The rapidly increasing transformation rate can be well described by exponential growth: when plotted on a logarithmic scale vs time, the ZPL intensity increase is linear. This behavior is typically observed also when reaction conditions are changed. There is always a delay time (induction period) before the formation of h-KRSF starts, and after that the ZPL intensity increases exponentially with time. The reaction conditions were varied by changing the Rb/K ratio and the alcohol used. The minimum fraction of Rb required to form the hexagonal phase is 20% for the synthesis procedure followed, resulting in part hexagonal and part cubic KRSF. For all the different alcohols used (from methanol to butanol), the formation of h-KRSF was observed. The induction period varied and was longer for a lower Rb content (Section S8).

**Figure 7:**
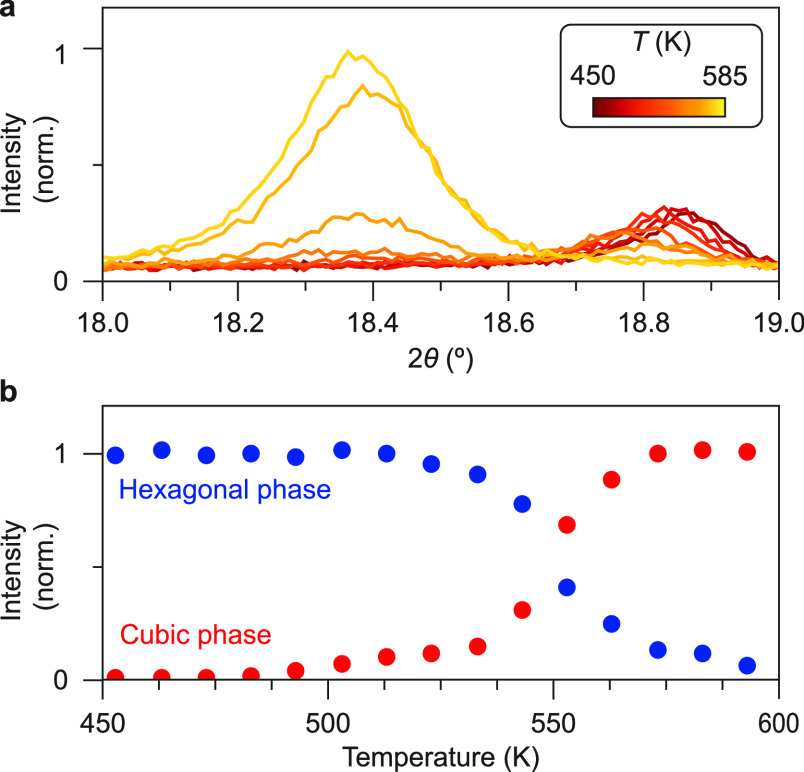
Monitoring the stability of h-KRSF with temperature-dependent XRD measurements. (a) Selection of diffractograms measured between 450 and 585 K. The peak at 18.85° corresponds to the hexagonal phase ((002) reflection), and the peak at 18.4° corresponds to the cubic phase ((111) reflection). (b) Integrated intensity underneath the peaks corresponding to the hexagonal (blue) and cubic (red) phase of KRSF with temperature intervals of 10 K. Note that for recording these diffractograms, a Cu K_α_ (λ_Kα_ = 1.5418 Å) X-ray source is used.

Before discussing the formation mechanism, it is good to evaluate the thermodynamic stability of the hexagonal vs cubic phase. To test this, first, temperature-dependent XRD was used. Diffractograms from 17.5 to 24.0° (2θ) were measured. This range was chosen because in this area there are peaks that distinctively belong to either the cubic or hexagonal KRSF. After each measurement, the sample was heated by 10 K, and the next diffractogram was measured. The results between 18 and 19° 2θ are shown in Figure 7, and the full pattern (17.5–24.0° 2θ) is shown in Section S9. Upon heating above 500 K, the peaks at 18.85 and 20.1° 2θ (from h-KRSF) diminish and then disappear, while the peaks at 18.4 and 21.2° 2θ (from c-KRSF) increase in intensity and then remain constant above 570 K. After cooling down to RT, the peaks at 18.85 and 20.1° 2θ do not reappear. Add that the pure h-KSF could be more stable and that a search for these materials could reduce thermal instability and sensitivity to moisture.

The transformation from hexagonal to cubic indicates that at higher temperatures, the cubic phase is the most stable phase. The observation that the peaks at 18.85 and 20.1° 2θ do not reappear upon cooling shows that the transformation is irreversible. Note that this is different from K_2_MnF_6_ for which the cubic phase is not stable at RT. Heating hexagonal K_2_MnF_6_ to 440 °C transformed the crystals to the cubic phase but after storing the crystals at room temperature they transformed back to the hexagonal phase.^29^ To test whether c-KRSF transforms back to h-KRSF at lower temperatures, several experiments were done: the material was kept for months at 253 K and RT, cubic material was heated for 1 month at 373 K and also heated and then slowly cooled from 573 to 435 K in 90 h. No XRD peaks of h-KRSF were found in any of the diffractograms recorded afterward indicating that c-KRSF is the stable phase around and above RT. The XRD results were confirmed by luminescence measurements, which showed no ZPL at 621.5 nm and only the emission spectra typical of c-KRSF.

Based on the observations so far, one can only speculate on the formation mechanism of h-KRSF. Initially, when the aqueous solution is added to the EtOH, nanosized c-KRSF particles are formed. Precipitation at the bottom of the beaker occurs gradually during evaporation. Possibly, the decreased alcohol content destabilizes the nanocrystals and induces particle growth. At the same time, it can destabilize the surface of the nanoparticles. It is known that differences in solvent changes the surface–solvent interaction and can affect the obtained crystal structure of polymorphic materials.^30,31^ A high surface area may induce the transformation to a structure with a higher density and thus less surface area.^32^ This can explain the transformation to h-KSF, as h-KSF has a higher reported density than c-KSF (2.87 vs 2.746 g/cm^3^).^6^ Once particles transform to the hexagonal phase, they can serve as seeds that grow at the expense of dissolving c-KRSF nanoparticles and give rise to an exponential increase of the fraction of h-KRSF over c-KRSF with time. A similar rapid increase in the conversion rate was recently observed by some of us in the transformation of cubic (α-phase) NaYF_4_ nanocrystals to larger hexagonal (β-phase) NaYF_4_ nanocrystals.^33^ We were able to model this transformation by taking into account a distribution in reaction (dissolution/growth) rates for nanoparticles, first resulting in a bimodal size distribution followed by an increasingly rapid transformation to large and monodisperse β-phase crystallites with time, similar to what is observed in Figure 6b.

To obtain better insight into and evidence for a formation mechanism, further studies, such as combined *in situ* WAXS and SAXS measurements, are required to follow particle size and crystallinity in time and relate these to the time-dependent luminescence properties. Indeed, also other mechanisms have been reported where an induction period is followed by a rapidly increasing transformation rate, for example, the transformation of ferrihydrite to goethite or hematite nanocrystals.^34^ Alternatively, autocatalysis can explain exponential growth of the phase transformation rate. This mechanism has been extensively studied, for example, the transformation of α- to β-Sn.^35^

A final challenge is the formation of hexagonal KSF free of Rb, especially since the presence of Rb can be linked to a lower stability of the phosphor under the extreme conditions experienced in wLEDs. To lower the amount of Rb, the synthesis of h-KRSF was done with different Rb/K ratios. Lowering the Rb-fraction resulted in longer induction periods and slower formation of h-KRSF. For 40% and 30% Rb, still a complete transformation to h-KRSF was observed. For 20% Rb, there was no complete transformation (for details, see Section S9), while for 10% and 0% Rb, no formation of h-K(R)SF could be observed (no increase in ZPL intensity). However, based on earlier reports on the synthesis of h-KSF by Kolditz in 1963 and Gossner in 1904^7,8^ and the observation of h-KSF in refs. (10) and (11), it is evident that h-KSF can be obtained, and it is worthwhile pursuing a synthesis method to realize the synthesis of h-KSF doped with Mn^4+^ with superior performance as a wLED phosphor.

To understand the role of Rb in the formation of h-KRSF, DFT calculations were done to determine the formation energies of cubic and hexagonal Rb_2_SiF_6_, KRbSiF_6_, and K_2_SiF_6_. The results and a more extensive discussion are provided in Section S10. In h-KRSF, there are (in contrast with c-KRSF) two nonequivalent M^+^ sites. The calculations show that the lowest energy configuration of h-KRSF is obtained when K^+^ ions occupy the smaller M1 site and Rb^+^ ions occupy the larger M2 site. If the ordering of the monovalent cations during crystal growth is indeed responsible for the formation of the (thermodynamically favorable) hexagonal phase, this could trigger the chain reaction among the other crystals we observe. Single-crystal XRD data could provide further information about the location of the Rb and K ions in the lattice and test this hypothesis. The role of K^+^ and Rb^+^ ordering could also play a role in the phase transition to the cubic phase at high temperature. Disorder induced by M^+^ ion migration can trigger the transformation to the cubic phase, which may be kinetically stable when, even for slow cooling back to room temperature, the ordering of K^+^ on M1 and Rb^+^ on M2 sites is hampered.

To quantify how stable the hexagonal phase is compared with the cubic phase, the formation energy of the hexagonal phase was subtracted from the cubic phase. The calculations show that in all cases, the cubic phase is more stable. However, it can be seen that K_2_SiF_6_ and Rb_2_SiF_6_ have a much stronger preference for the cubic phase as the energy difference between the cubic and the hexagonal phases is 43 and 71 meV per unit cell, respectively, while it is only 9 meV for KRbSiF_6_. This confirms that for the mixed K/Rb composition, it is easier to form the hexagonal phase.

## Conclusion

The luminescence of Mn^4+^ in a new hexagonal phase of KRbSiF_6_ is reported. The optical properties have clear advantages over those for Mn^4+^ in the cubic KSF. The deviation from the inversion symmetry allows for the observation of a strong zero-phonon line and shortens the luminescence lifetime for Mn^4+^. This improves the lumen/W efficacy, increases the absorption strength, and reduces saturation at high blue photon fluxes. The quenching temperature of the Mn^4+^ luminescence in the hexagonal phase is very high and comparable to that in the cubic phase (>500 K). High quantum yields (>90%) are realized without synthesis optimization but the stability is lower, probably due to the large fraction of Rb. The h-KRSF is synthesized by adding precursors dissolved in water to an excess volume of ethanol followed by slow evaporation of the ethanol. The formation mechanism is intriguing and was studied by continuously measuring luminescence spectra of the (nano)particles in the reaction volume. After an induction period of ∼15 h, the precipitate started to transform to the hexagonal phase with an exponentially increasing transformation rate. After 8 h, it was fully transformed. The stability of the hexagonal phase was tested by temperature-dependent XRD and luminescence measurements showed that above 200°C, h-KRSF transforms back to c-KRSF. The higher efficacy, shorter luminescence lifetime, and high quenching temperature make the hexagonal phase superior to cubic KSF, especially if a Rb-free synthesis route for pure h-KSF can be found to match the stability of c-KSF.

## Supporting Information Available

Luminous efficacy calculations, incorporation in wLED, XRD patterns as a function of [Mn^4+^], ICP-OES, temperature-dependent emission and XRD, *in situ* emission setup and ab initio DFT calculations (PDF)

## References

[ref1] Paulusz A. G. Efficient Mn(IV) Emission in Fluorine Coordination. J. Electrochem. Soc. 1973, 120 (7), 942 10.1149/1.2403605.

[ref2] Radkov E. V.; Grigorov L. S.; Setlur A. A.; Srivastava A. M. Red Line Emitting Phosphors for Use in Led Applications. US Patent US 7,497,973 B2, 2010.

[ref3] Yan S. Critical Review—On the Anomalous Thermal Quenching of Mn^4+^ Luminescence in A_2_ XF_6_:Mn^4+^ (A = K, Na, Rb or Cs; X = Si, Ti, Ge, Sn, Zr or Hf).. ECS J. Solid State Sci. Technol. 2020, 9 (10), 106004 10.1149/2162-8777/abc512.

[ref4] Xu Y. K.; Adachi S. Properties of Na_2_SiF_6_:Mn^4+^ and Na_2_GeF_6_:Mn^4+^ Red Phosphors Synthesized by Wet Chemical Etching. J. Appl. Phys. 2009, 105 (1), 013525 10.1063/1.3056375.

[ref5] Adachi S. Photoluminescence Spectra and Modeling Analyses of Mn^4+^-Activated Fluoride Phosphors: A Review. J. Lumin. 2018, 197, 119–130. 10.1016/j.jlumin.2018.01.016.

[ref6] Adachi S. Review—Temperature Dependence of Transition-Metal and Rare-Earth Ion Luminescence (Mn^4+^, Cr^3+^, Mn^2+^, Eu^2+^, Eu^3+^, Tb^3+^, Etc.) I: Fundamental Principles. ECS J. Solid State Sci. Technol. 2022, 11 (9), 096002 10.1149/2162-8777/ac8bf8.

[ref7] Kolditz L.; Preiss H. Über Fluorhaltige Komplexe. VII. Über Alkoxysilicate Und Fluorosilicate. Z. Anorg. Allg. Chem. 1963, 325 (5–6), 245–251. 10.1002/zaac.19633250504.

[ref8] Gossner B. VIII. Untersuchung Polymorpher Körper. Z. Kristallogr. - Cryst. Mater. 1904, 38 (1–6), 110–168. 10.1524/zkri.1904.38.1.110.

[ref9] Gramaccioli C. M.; Campostrini I. Demartinite, a New Polymorph of K_2_SiF_6_ from La Fossa Crater, Vulcano, Aeolian Islands, Italy. Can. Mineral. 2007, 45 (5), 1275–1280. 10.2113/gscanmin.45.5.1275.

[ref10] Denaeyer M.-E.; Ledent D. Sur La Présence de La Modification Hexagonale de La Hiératite (Camermanite) Dans Des Incrustations de Sels Potassiques d’une Cheminée d’usine. Bull. Soc. Fr. Mineral. Cristallogr. 1952, 75 (4), 231–236. 10.3406/bulmi.1952.4772.

[ref11] Lazarowska A.; Mahlik S.; Grinberg M.; Lin C. C.; Liu R.-S. Pressure Effect on the Zero-Phonon Line Emission of Mn^4+^ in K_2_SiF_6_. J. Chem. Phys. 2015, 143 (13), 134704 10.1063/1.4932181. 26450325

[ref12] Lv L.; Jiang X.; Huang S.; Chen X.; Pan Y. The Formation Mechanism, Improved Photoluminescence and LED Applications of Red Phosphor K_2_SiF_6_:Mn^4+.^. J. Mater. Chem. C 2014, 2 (20), 3879–3884. 10.1039/C4TC00087K.

[ref13] Weiler V.; Schmidt P. J.; Schnick W.; Seibald M. A. Mn-Activated Hexafluorosolicates for LED Applications. US 9,422,471 B2, 2014.

[ref14] Efficient Preparations of Fluorine Compounds; Roesky H. W., Ed.; John Wiley & Sons, 2013.

[ref15] Bockstedte M.; Kley A.; Neugebauer J.; Scheffler M. Density-Functional Theory Calculations for Poly-Atomic Systems: Electronic Structure, Static and Elastic Properties and Ab Initio Molecular Dynamics. Comput. Phys. Commun. 1997, 107 (1–3), 187–222. 10.1016/S0010-4655(97)00117-3.

[ref16] Nityananda R.; Hohenberg P.; Kohn W. Inhomogeneous Electron Gas. Resonance 2017, 22 (8), 809–811. 10.1007/s12045-017-0529-3.

[ref17] Kohn W.; Sham L. J. Self-Consistent Equations Including Exchange and Correlation Effects. Phys. Rev. 1965, 140 (4A), A1133 10.1103/PhysRev.140.A1133.

[ref18] Kresse G.; Joubert D. From Ultrasoft Pseudopotentials to the Projector Augmented-Wave Method. Phys. Rev. B: Condens. Matter Mater. Phys. 1999, 59 (3), 1758 10.1103/PhysRevB.59.1758.

[ref19] Kresse G.; Furthmüller J. Efficient Iterative Schemes for Ab Initio Total-Energy Calculations Using a Plane-Wave Basis Set. Phys. Rev. B: Condens. Matter Mater. Phys. 1996, 54 (16), 11169–11186. 10.1103/PhysRevB.54.11169. 9984901

[ref20] Perdew J. P.; Burke K.; Ernzerhof M. Generalized Gradient Approximation Made Simple. Phys. Rev. Lett. 1996, 77 (18), 3865–3868. 10.1103/PhysRevLett.77.3865. 10062328

[ref21] Loehlin J. H. Redetermination of the Structure of Potassium Hexafluorosilicate, K_2_SiF_6_. Acta Crystallogr., Sect. C: Struct. Chem. 1984, 40 (3), 570–570. 10.1107/S0108270184004893.

[ref22] Rienmüller J.; Bandemehr J.; Kraus F. Single-Crystal Structures of A_2_SiF_6_ (A = Tl, Rb, Cs), a Better Structure Model for Tl_3_[SiF_6_]F, and Its Novel Tetragonal Polymorph. Z. Naturforsch., B: J. Chem. Sci. 2021, 76 (10–12), 559–565. 10.1515/znb-2021-0024.

[ref23] Shannon R. D. Revised Effective Ionic Radii and Systematic Studies of Interatomic Distances in Halides and Chalcogenides. Acta Crystallogr., Sect. A: Found. Adv. 1976, 32 (5), 751–767. 10.1107/S0567739476001551.

[ref24] de Jong B. H. W. S.; Supèr H. T. J.; Frijhoff R. M.; Spek A. L.; Nachtegaal G. Mixed Alkali Systems: Dietzel’s Theorem, X-Ray Structure, Hygroscopicity, and 29-Si MAS NMR of NaRbSi_2_O_5_ and NaCsSi_2_O_5_. Z. Kristallogr. - Cryst. Mater. 2000, 215 (7), 397–405. 10.1524/zkri.2000.215.7.397.

[ref25] Nguyen H.-D.; Lin C. C.; Liu R.-S. Waterproof Alkyl Phosphate Coated Fluoride Phosphors for Optoelectronic Materials. Angew. Chem., Int. Ed. Engl. 2015, 54 (37), 10862–10866. 10.1002/anie.201504791. 26214154

[ref26] Zhou Y.-Y.; Song E.-H.; Deng T.-T.; Zhang Q.-Y. Waterproof Narrow-Band Fluoride Red Phosphor K_2_TiF_6_:Mn^4+^ via Facile Superhydrophobic Surface Modification. ACS Appl. Mater. Interfaces 2018, 10 (1), 880–889. 10.1021/acsami.7b15503. 29211450

[ref27] Nguyen H.-D.; Liu R.-S. Narrow-Band Red-Emitting Mn^4+^-Doped Hexafluoride Phosphors: Synthesis, Optoelectronic Properties, and Applications in White Light-Emitting Diodes. J. Mater. Chem. C 2016, 4 (46), 10759–10775. 10.1039/C6TC03292C.

[ref28] Senden T.; van Dijk-Moes R. J. A.; Meijerink A. Quenching of the Red Mn^4+^ Luminescence in Mn^4+^-Doped Fluoride LED Phosphors. Light: Sci. Appl. 2018, 7 (1), 8 10.1038/s41377-018-0013-1. 30839606 PMC6106983

[ref29] Sijbom H. F.; Verstraete R.; Joos J. J.; Poelman D.; Smet P. F. K_2_SiF_6_:Mn^4+^ as a Red Phosphor for Displays and Warm-White LEDs: A Review of Properties and Perspectives. Opt. Mater. Express 2017, 7 (9), 3332–3365. 10.1364/OME.7.003332.

[ref30] Chu H.; Li X.; Chen G.; Zhou W.; Zhang Y.; Jin Z.; Xu J.; Li Y. Shape-Controlled Synthesis of CdS Nanocrystals in Mixed Solvents. Cryst. Growth Des. 2005, 5 (5), 1801–1806. 10.1021/cg050068w.

[ref31] Stoica C.; Verwer P.; Meekes H.; van Hoof P. J. C. M.; Kaspersen F. M.; Vlieg E. Understanding the Effect of a Solvent on the Crystal Habit. Cryst. Growth Des. 2004, 4 (4), 765–768. 10.1021/cg0342314.

[ref32] Croker D.; Hodnett B. K. Mechanistic Features of Polymorphic Transformations: The Role of Surfaces. Cryst. Growth Des. 2010, 10 (6), 2806–2816. 10.1021/cg901594c.

[ref33] Prins P. T.; van der Bok J. C.; van Swieten T. P.; Hinterding S. O. M.; Smith A. J.; Petukhov A. V.; Meijerink A.; Rabouw F. T. The Formation of NaYF_4_:Er^3+^, Yb^3+^ Nanocrystals Studied by In Situ X-Ray Scattering: Phase Transition and Size Focusing. Angew. Chem., Int. Ed. 2023, 62 (28), e202305086 10.1002/anie.202305086. 37170964

[ref34] Das S.; Hendry M. J.; Essilfie-Dughan J. Transformation of Two-Line Ferrihydrite to Goethite and Hematite as a Function of PH and Temperature. Environ. Sci. Technol. 2011, 45 (1), 268–275. 10.1021/es101903y. 21128633

[ref35] Cornelius B.; Treivish S.; Rosenthal Y.; Pecht M. The Phenomenon of Tin Pest: A Review. Microelectron. Reliab. 2017, 79, 175–192. 10.1016/j.microrel.2017.10.030.

