# Supplementary material for: Luminescence and Formation of Cubic and Hexagonal (K,Rb)2SiF6:Mn4+
Source: ACS Appl Mater Interfaces. 2023 Dec 18;16(1):1044–53. doi: 10.1021/acsami.3c13715 (PMC10788833; doi:10.1021/acsami.3c13715)
Supplement: Supplementary file 1 — am3c13715_si_001.pdf [file am3c13715_si_001.pdf]

Supporting Information

Luminescence and formation of cubic and hexagonal $(K,Rb)_2SiF_6$: Mn^{4+}

Arnoldus J. van Bunningen[1†], *Jur W. de Wit*[1†], *Sadakazu Wakui*[2] *and Andries Meijerink*[1*]

[1]*Debye Institute for Nanomaterials Science, Utrecht University, Princetonplein 1, 3584 CC Utrecht, The Netherlands,*
[2]*Nichia Corporation, 491 Oka, Kaminaka-Cho, Anan-Shi, Tokushima 774-8601, Japan*

[†] These authors contributed equally
[*] Corresponding author. E-mail: a.meijerink@uu.nl

Contents

Section S1. Luminous efficacy and absorption increase
Section S2. Performance of h-KRSF in a wLED phsophor
Section S3. XRD patterns KRSF:Mn^{4+} with different [Mn^{4+}]
Section S4. Determination of [K], [Rb] and [Mn] with ICP-OES and incorporation rate of [Mn^{4+}]
Section S5. Emission intensity as a function of [Mn^{4+}] – undiluted and diluted with $BaSO_4$
Section S6. Temperature-dependent emission spectra
Section S7. Experimental set-up *in-situ* emission
Section S8. Influence of K/Rb ratio and choice of antisolvent
Section S9. Temperature-dependent XRD measurements
Section S10. *Ab-initio* DFT calculations on phase stability in hexagonal and cubic KSF, KRSF and RSF

Section S1. Luminous efficacy and absorption increase

The luminous efficacy (Lm/W) of cubic KRSF, hexagonal KRSF, and cubic KSF are calculated and compared with each other. The spectra of these materials are shown in figure S2 and are normalized with respect to their integral. In the same figure the photopic eye responsivity curve is plotted in black, highlighting the advantage of Mn^{4+}-doped fluorides and hexagonal KRSF:Mn^{4+} in particular. In the CCD spectrometer and PMT detectors used in our research, the number of counts as a function of wavelength is proportional to the number of photons that are registered by the detector. As energy is proportional to 1/λ the measured intensity needs to be converted into energy by dividing all measured intensities by its corresponding wavelength. With the corrected spectrum, the luminous efficacy of the Mn-doped fluorides is calculated between 580 and 680 nm:

$$\text{Luminous Efficacy} = \frac{\int_{580}^{680} V(\lambda)\Phi_\lambda d\lambda}{\int_{580}^{680} \Phi_\lambda d\lambda} \tag{1}$$

Where $V(\lambda)$ is the eye responsivity curve and Φ_λ is the radiant flux in W/nm. The numerator of the fraction describes the luminous flux and the denominator describes the radiant flux. Based on this calculation the luminous efficacy of hexagonal KRSF is determined to be 1.2 % higher than for c-KSF and 2.9 % higher than for c-KRSF.

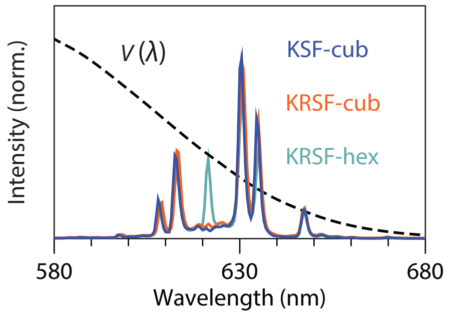

Figure S1 | Normalized emission spectra of cubic KSF, cubic KRSF and hexagonal KRSF plotted with the eye reponsivity curve *V* (λ). Because the emission redshifts a little going from KSF to KRSF, the luminous efficacy improvement is slightly larger for h-KRSF to c-KRSF than for h-KRSF to c-KSF.

In order to quantify the increase in absorption strength caused by the relaxation of the parity selection rule, we measure the room temperature emission spectrum of h-KRSF and c-KRSF. The c-KRSF used for these measurements is obtained by heating h-KRSF to 698 K for 15 min, after which it will be fully transformed into c-KRSF. In terms of measurement conditions, there are no changes between the c-KRSF and h-KRSF measurement. We assume that the QY of both the cubic and hexagonal KRSF are the same after the phase transition. Because the h-KRSF will inherently emit more photons in the wavelength region of 580 to 680 nm, we omit the area under the ZPL peak (518 to 526 nm). Dividing the resulting integral values shows an increase in the absorption strength at 450 nm of 34% going from c-KRSF to h-KRSF.

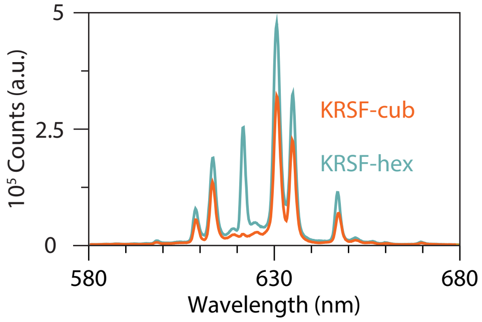

Figure S2 | Room temperature emission spectra of hexagonal KRSF and cubic KRSF formed after heating h-KRSF. After heating the h-KRSF to 698 K and cooling it down to RT, it has fully transformed into c-KRSF. A comparison of the integral of the two emission spectra reveals that (assuming the same QY) the absorption strength of the h-KRSF is 34% higher than that of the c-KRSF.

Section S2. Performance of h-KRSF in a wLED phsophor

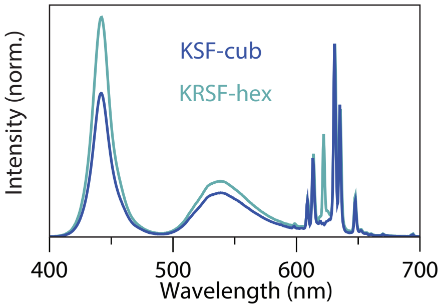

Figure S3 | Comparison of wLED performance of a blue LED coated with YAG:Ce^{3+} and KSF or KRSF. Both LED spectra are normalized with respect to the most intense ν_6 emission peak around 630 nm. Note the additional ZPL peak around 620 nm for the hex-KRSF sample.

Section S3. XRD patterns KRSF:Mn^{4+} with different [Mn^{4+}]

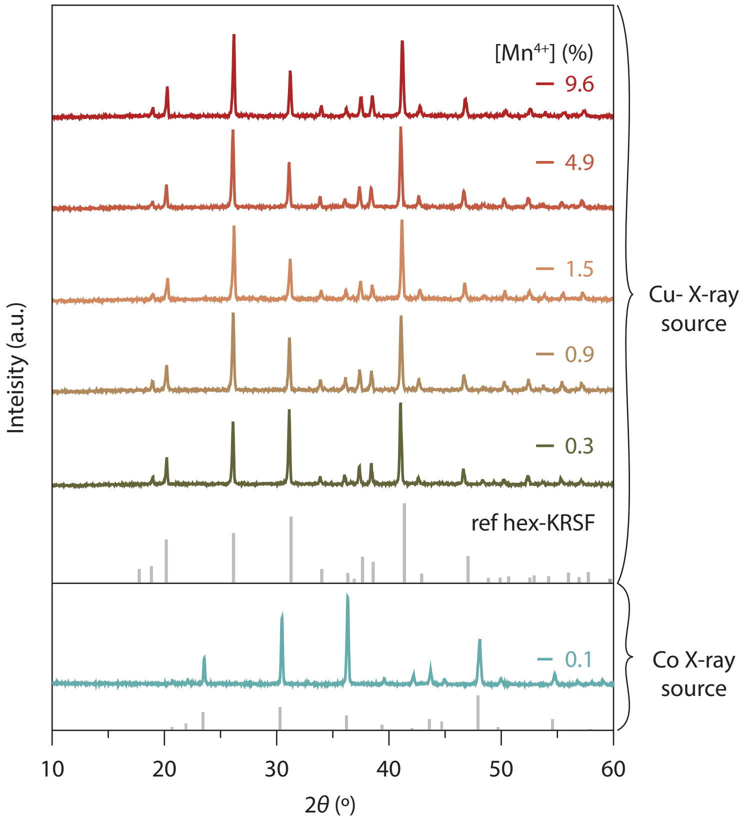

Figure S4 | XRD patterns for KRbSi$_{(100-x)/100}$Mn$_{(x/100)}$F$_6$: x% Mn^{4+}). The patterns were recorded with the Malvern Panalytical Aeris Research diffractometer and a Cu Kα ($\lambda_{K\alpha}$ = 1.5418 Å) radiation source. The Mn^{4+} concentrations are determined with ICP-OES, exact concentrations of K, Rb and Mn can be found in Section S4.

Section S4. Determination of [K], [Rb] and [Mn] with ICP-OES and incorporation rate of [Mn^{4+}]

The concentration of Mn^{4+} in the KRSF host material is determined with respect to the combined K^+ and Rb^+ concentration. Because these three elements were all present in a single multi-element calibration solution, only one calibration curve had to be prepared. Therefore, the (K+Rb)/Mn ratio was used to determine the actual doping concentration of Mn^{4+}. The results are are presented in **table S1**. Note that when a high [Mn^{4+}] of 40% was used, the actual incorporated [Mn^{4+}] was only 6.49 %. Because of the lower incorporated Mn^{4+} concentration than for the 20% doped sample, the sample was omitted from the concentration-dependent measurement series.

Feed [Mn] (%)	[K]	[Rb]	[Mn] (%)
0.5	1.10	0.90	0.13
1	1.14	0.86	0.14
2	1.17	0.83	0.27
5	1.26	0.74	0.90
7	1.27	0.73	1.50
10	1.29	0.71	4.86
20	1.43	0.57	9.61
40	1.62	0.38	6.49

Table S1 | The concentration of K, Rb and Mn in hexgonal $K_2 - y Rb_y Si_{(100-x)/100} Mn_{(x/100)} F_6$: x% Mn^{4+} as determined with ICP-OES. The concentration of K, Rb and Mn are determined at λ_{em}=766.490 nm, λ_{em}=780.023 nm and λ_{em}=257.610 nm respectively. Note that the concentration of K increases with increasing Mn doping concentrations because the Mn precursor is K_2MnF_6.

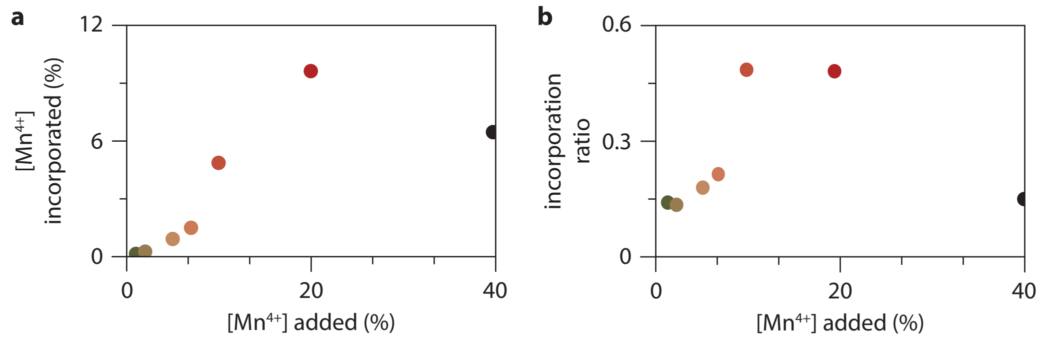

Figure S5 | Incorporation of Mn^{4+} in h-KRSF. (a) Incorporation into the host lattice as a function of feed concentration. **(b)** Incorporation ratio as a function of feed concentration. The black datapoint represents the sample with a 40% feed [Mn^{4+}], which results in an actual incorporated [Mn^{4+}] of 6.49%.

Section S5. Emission intensity as a function of $[Mn^{4+}]$ – diluted and undiluted

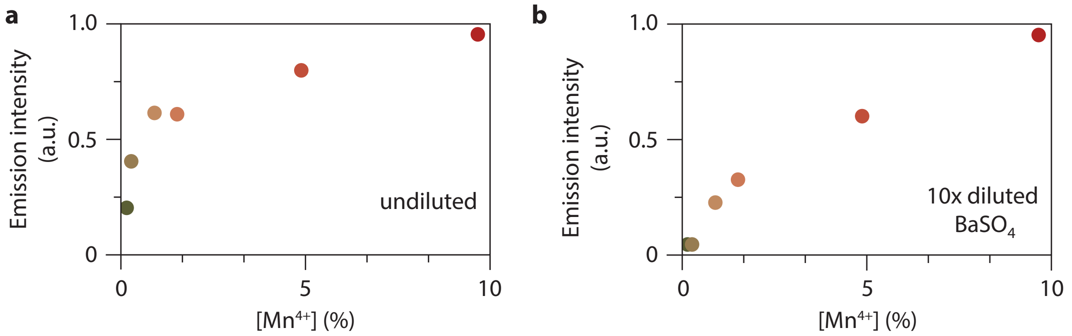

Figure S6 |Emission intensity as a function of $[Mn^{4+}]$. (a) Integrated emission intensity between 580–680 nm as a function of $[Mn^{4+}]$ in KRSF. **(b)** Same as in panel **a**, but for samples that are diluted (weight dilution) 10× with $BaSO_4$ in order to reduce the effect of blue light exctinction on the measured emission intensity. Even though in both measurements the emission intensity does not increase linearly as a function of $[Mn^{4+}]$, the behavior becomes much more linear by diluting the samples. This observation, in addition to the constant lifetime as a function of $[Mn^{4+}]$ **(figure 4d in the main text)** shows that concentration quenching does not play an important role up to 10 % Mn^{4+}.

Section S6. Temperature-dependent emission spectra

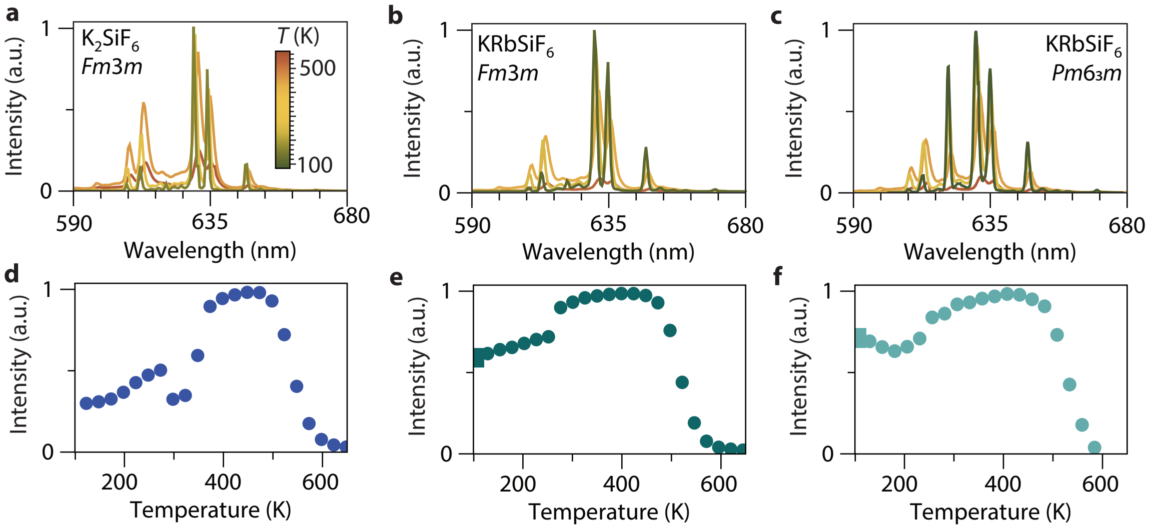

Figure S7 | Temperature-dependent emission spectra and integrated emission intensities for cubic KSF, cubic KRSF and hexagonal KRSF between 100 and 650 K. (a–c) A selection of emission spectra between 100 and 600 K for all three samples. **(d–f)** Integrated intensity of the spectra between 590 and 680 nm. Note that the temperature range for hexagonal KRSF does not extend beyond 600 K, as it is fully converted into cubic KRSF at that temperature.

Section S7. Experimental set-up *in-situ* emission

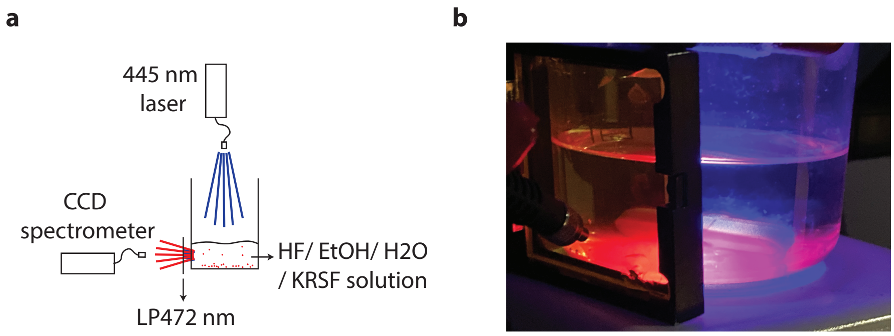

Figure S8 | Schematic and photographic depiction of the experimental set-up for tracking the growth of hexagonal KRSF. (a) Schematic depiction of the optical set-up. **(b)** photograph of the set-up with the fiber-coupled spectrometer visible on the left hand side, after the 472nm LP filter.

Section S8. Influence of K/Rb ratio and other investigated parameters infuencing the h-KRSF phase formation

We have attempted to synthesize pure h-KSF by reducing the Rb concentration. However, below 20% Rb concentration no phase pure h-KRSF was obtained using the synthesis protocol described in the methods section. With the 20% Rb sample, we obtained mixed phase h-KRSF and c-KRSF (**figure S9a**). The 20% Rb sample was therefore used to check which parameters tip the reaction in favour of obtaining phase pure h-KRSF.

The first investigated parameter is the amount of EtOH used in the synthesis. Using double the amount of EtOH does result in phase pure h-KRSF for the 20% Rb sample (**figure S9b**). The reaction took much longer to complete for the 20% Rb sample. The excess EtOH probably facilitates a prolonged reaction time, because of which the full conversion can take place. To further investigate the influence of reaction time, we used the same 20% Rb sample and tracked the growth in different antisolvents: MeOH, EtOH, extra EtOH, iso-PrOH and BuOH. We monitored these experiments in-situ by tracking de rise of the ZPL peak intensity at 4 points in time (**figure S9c**). The green line indicates the synthesis with extra EtOH and its ZPL intensity indicates a full conversion to h-KRSF. It is noteworthy that the full conversion in extra EtOH takes much longer for the for the 20% Rb synthesis (∼120h) than for the 50% Rb sample (∼8h). Indicating that an equal presence of K and Rb increases the rate of the autocatalytic process. For the other alcohols, the amount of antisolvent was 200 ml, like in the standard synthesis. Even though the evaporation rate decreased (BP_{MeOH} = 78°C and BP_{BuOH} = 117°C), the prolonged reaction time did not result in full conversion to h-KRSF in e.g. iso-PrOH and BuOH. Possibly the fact that these liquids are not as miscible with water plays a role. It could be interesting to further investigate the role of the liquid evaporation by (partly) covering the reaction beaker.

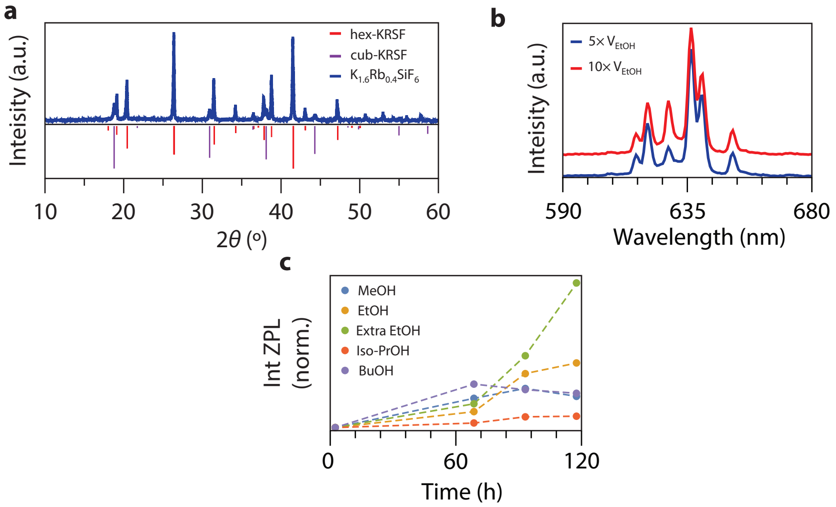

Figure S9 | Influence of the K/Rb ratio and the choice of antisolvent on the formation of h-KRSF. (a) XRD pattern of $K_{1.6}Rb_{0.4}SiF_6$ doped with 0.1% Mn^{4+}, synthesized with the protocol described in the methods section. The XRD pattern matches with both the cubic and hexagonal phase of KRSF. **(b)** Upon pouring the K/Rb=0.2 reaction mixture into extra EtOH, the full cubic-to-hexagonal transformation takes place again (red spectrum). Using the "standard" amount of EtOH does not result in a full conversion (blue spectrum). **(c)** The conversion from the cubic to the hexgonal phase for $K_{1.6}Rb_{0.4}SiF_6$ takes place over a time period that is roughly 5 times longer than that of the K/Rb=1 synthesis. If the regular amount (5x excess) of EtOH is used no full conversion is obtained. The effect of other alcohols was investigated as well. All tested alchohols other than EtOH performed worse in terms of the cubic-to-hexagonal phase transformation for the $K_{1.6}Rb_{0.4}SiF_6$.

Some other systems that show a phase transformation at a certain temperature, are induced by the presence of nucleation points.[1] A notable example is the conversion from β-Sn to α-Sn below −13 °C.[2] In this material α-Sn seeds are the nucleation points for the phase transformation. In our synthesis this nucleation point could be the hexagonal Mn^{4+} precursor $K_2MnF_6$6. However, upon doing a $K_1Rb_1SiF_6$ synthesis without adding hexagonal K_2MnF_6, the pure h-KRSF phase still forms.

Section S9. Temperature-dependent XRD measurements

In addition to the optical measurements, temperature-dependent XRD measurement are done to confirm the hexagonal-to-cubic phase transformation (**figure S10**).

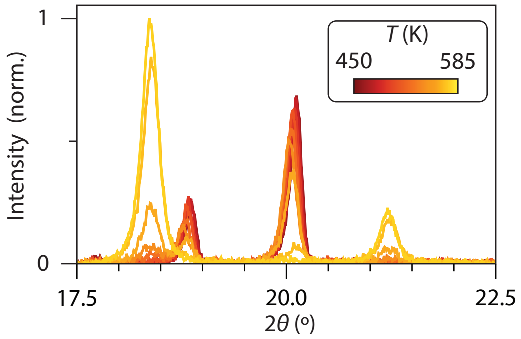

Figure S10 | Full range T-dependent XRD pattern. The peak intensity values shown in **figure 7b** in the main text are determined based on both the cubic and the hexagonal peaks in this pattern.

Section S10. *Ab-initio* DFT calculations on phase stability in hexagonal and cubic KSF, KRSF and RSF

DFT calulations were performed to calculate the formation energies of different crystal structures. For h-KRSF there are 4 positions for K/Rb within the unit cell, 2 smaller M1 (2b) sites and 2 larger M2 (2a) sites. The fractional coordinates of the sites along with their symmetry are shown in **table S2a**. Of these positions two are occupied by K and two by Rb in our calculations. The number of possible placements is ${}^4C_2$=6, which can be reduced to 4 because of the symmetry. For h-KRSF, 4 calculations are performed with different occupations of the 4 sites. The lowest energy configuration is obtained when K occupies the smaller M1 sites and Rb the larger M2 sites. The energy of this configuration is chosen as the energy of h-KRSF (**table S2b**).

a

site	a1	a2	a3	wyck. symb.
1	2/3	1/3	0.0875	2b
2	1/3	2/3	0.5875	2b
3	0	0	0.8122	2a
4	0	0	0.3122	2a

b

Configuration on site 1, 2, 3, 4		E_{tot} (eV)
Rb, Rb, K, K		−92.8130
K, K, Rb, Rb		**−93.0058**
Rb, K, Rb, K	(K, Rb, K, Rb)	−92.9090
K, Rb, Rb, K	(Rb, K, K, Rb)	−92.9382

Table S2 | Atomic positions of M^+ sites in h-KRSF and total energies at different K^+ and Rb^+ placements. (**a**) Fractional coordinates of the different sites within the primitive cell for h-KRSF. Note that the wyckoff symbols are different, indicating a different coordinating polyhedron.(**b**) Total energy of h-KRSF for different K an Rb configurations. The energy (bold) for the K, K, Rb, Rb placement is lowest and that there are two energetically equivalent placements possible (bottom rows).

The DFT calculations indicate an ordering in the h-KRSF crystal where the small M1 site is occupied by K and the larger M2 site by Rb. In the cubic phase, there is only one M1 site. It could be that the placement of the Rb and the K on the large and small site respectively is the driving force of the hexagonal phase transformation. This thermodynamically more stable phase is never obtained under alternative (faster) synthesis conditions because of the kinetically favoured random placements of cations.

Our hypothesis is that as the sample heats up (above 200°C), this ordering is lost and the h-KRSF reverts to the cubic phase. As mentioned in the main text, we have performed a few control measurements to check if the hexagonal phase could be obtained from the cubic phase after it had formed:

- Cool c-KRSF to 253 K for 2 months
- Heat c-KRSF to 373 K for 3 months
- Heat c-KRSF to 573 K and cool down to 435 K over the course of 90 hours

All these experiments failed in obtaining h-KRSF, unfortunately neither confirming nor disproving the hypothesis that the ordering of the K and Rb ions is indeed responsible for the formation of the hexagonal phase. Single crystal XRD measurements on h-KRSF could provide valuable further insights on the ordering of K and Rb ions in the lattice.

Finally, we compared the formation energies of cubic KSF, KRSF and RSF with their hexagonal and trigonal counterparts. The results are shown in **figure S11**. The primitive unit cell is used for the calculation of cubic phase. The primitive vectors are (0,a/2,a/2), (a/2,0,a/2), (a/2,a/2,0). In the case of the cubic cell, there are 2 equivalent positions for K, where one is for K and the other for Rb. So, for c-KRSF only one structure is calculated. The calculated formation energies of each cubic compound are subtracted by the formation energies of the hexagonal compound. The resulting energy difference is in all cases negative (favourable to form cubic MSF), but smallest for h-KRSF.

	Fm-3m	P63mc	P-3m1	*E* (Fm-3m – P63mc)
KSF	−46.6430	−46.6003	−46.5225	−0.043
KRSF	−46.5118	−46.5029	-	−0.009
RSF	−46.4435	−46.3724	−46.2780	−0.071

Values in eV.

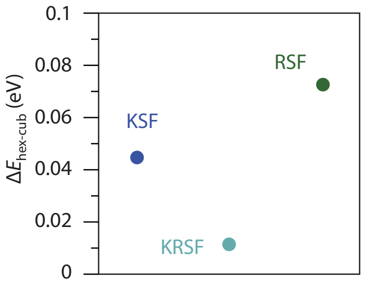

Figure S11 | Results for *ab-initio* DFT calculations for the formation energy per unit cell of cubic, hexagonal and trigonal KSF, KRSF and RSF. Note that the total energy of the h-KRSF is halved with respect to the values in **table S2** because there 2 unit cells are used in the calculations.

References

1. Cardew, P. T., ; Davey, R. J. The kinetics of solvent-mediated phase transformations. *Proc. R. Soc. A: Math. Phys. Eng. Sci.* **1985** 398(1815), 415-428.

2. Cornelius, B.; Treivish, S.; Rosenthal, Y.; Pecht, M. The Phenomenon of Tin Pest: A Review. *Microelectron. Reliab.* **2017**, 79, 175–192.
